# Massive open online courses (MOOCs) in genomic variant interpretation: An innovative education strategy for the growing genetic counselor workforce

**DOI:** 10.1002/jgc4.1837

**Published:** 2023-11-27

**Authors:** Beth Coad, Katherine Joekes, Alicja Rudnicka, Amy Frost, Katrina Tatton‐Brown, Katie Snape

**Affiliations:** ^1^ St George's University of London London UK; ^2^ Genomics Education Programme, Health Education England London UK

**Keywords:** education, genetic counselor, MOOCs, variant classification

## Abstract

The growth in genomic testing in healthcare requires a highly trained specialist workforce to ensure evidence based clinical germline variant interpretation. Genetic counselors form a core part of the clinical genomics multidisciplinary team (MDT) and represent a growing workforce participating in variant interpretation from data analysis to the patient consultation. Standardized, high‐quality variant interpretation training for Genetic Counselors has historically been ad hoc and variable, with existing programs lacking capacity to reach the entire workforce. To address the requirement for scalable variant interpretation training for genomics healthcare professionals (HCPs), two Massive Open Online Courses (MOOCs) were developed. We analyzed the data from 17 Genetic counselors, as part of an evaluation cohort completing the first run of these MOOCs. Overall genetic counselors enjoyed the courses, felt they were clinically relevant and would recommend them to colleagues. Common challenges amongst the genetic counseling workforces included utilizing relevant databases and finding time in the workday to complete training. These findings suggest MOOCs could be an acceptable option to ensure a consistent and transferrable high standard of training, complimentary to existing curricula. They also hold the potential to facilitate large‐scale education to update the genetic counseling workforce when changes in variant interpretation guidance occur.

**What is known about this topic**

Minimal literature regarding the use of MOOCs as education innovation for the genetic counseling workforce, or for training in variant interpretation is available. Broader literature on MOOCs as an intervention in HCP education in other areas is available.

**What this paper adds to the topic**

This paper adds the considerations for MOOCs in training the genetic counseling workforce in variant interpretation.

## 1 INTRODUCTION

To truly harness the potential of genomic sequencing for patients, robust clinical variant interpretation is crucial. This necessitates a multidisciplinary team (MDT) approach requiring highly trained genomics specialists, including both clinicians and laboratory scientists. The role of genetic counselors in this MDT is widely recognized and varies internationally (Dwarte et al., 2019; Kohut et al., 2019; Middleton et al., 2022; Patch & Middleton, 2018; Wain et al., 2020). Genetic counselors may conduct bioinformatic analysis (Wain et al., 2020), contribute clinical expertise to MDT discussion (Dwarte et al., 2019; Kohut et al., 2019), explain variant of uncertain significance (VUS) results to patients (Dwarte et al., 2019; Kohut et al., 2019; Patch & Middleton, 2018), and train nongenomics specialist healthcare professionals (HCPs) in variant interpretation (Dwarte et al., 2019; Middleton et al., 2022).

This recognition of germline variant interpretation as a core part of genomic counseling practice is evidenced within the training curricula for genetic counselors, which includes bioinformatic training and laboratory attachments (Grove et al., 2019; HEE, 2022). In the United Kingdom, there have also been face‐to‐face taught courses available to genetic counselors to upskill in variant interpretation (AGNC, 2020; Wellcome, 2020). However, reliance on this resource heavy training delivered in silos may not be a sustainable solution to meeting the training need of the expanding genetic counselor workforce. In addition, the evolving nature of variant interpretation guidance means genetic counselors will require regular refresher training which is responsive to the constant developments in this field.

Here we present an innovative approach to meet this training need through the delivery of two massive open online courses (MOOCs).

## 2 MASSIVE OPEN ONLINE COURSES (MOOCs)

MOOCs are openly accessible asynchronous online courses which are used widely in healthcare (Liyanagunawardena & Williams, 2014), but are a novel tool for educating the genetic counseling workforce. Currently no other MOOCs in clinical genomic variant interpretation, and few considering education in other areas of genomics for genetic counselors, are available across popular MOOC platforms FutureLearn and Coursera (Coursera; FutureLearn, 2022).

These courses have grown in popularity across healthcare due to their broad reach and high level of learner flexibility when compared to in‐person teaching (Liyanagunawardena & Williams, 2014; Longhini et al., 2021). As well as being free for learners, they can also prove to be a cost‐effective method of meeting education needs, as while significant development costs apply (Maxwell et al., 2018), MOOCs do not require venue hire nor travel and can reach high numbers of learners through multiple runs of the same content (Setia et al., 2019). Studies often report high levels of learner satisfaction and show improvements in confidence amongst HCPs (Hoedebecke et al., 2018; Liyanagunawardena & Williams, 2014; Longhini et al., 2021; Pham et al., 2021). However, there is limited data surrounding the impacts of MOOC learning on clinical practice, as is similar amongst many healthcare education interventions (Rowe et al., 2019).

When first developed, MOOCs were freely available educational content developed by online communities. Increasing numbers of single source MOOCs in healthcare settings now have associated costs to the learner, such as subscription for lifetime access or fees to gain formal certification (FutureLearn, 2022; Maxwell et al., 2018). Despite this development, many healthcare MOOCs still aim to foster an atmosphere of community, incorporating interactive and social learning through online activities, quizzes and discussion boards (Liyanagunawardena & Williams, 2014; Maxwell et al., 2018). Despite these efforts to engage the learner, MOOCs have been criticized for high attrition rates, with reasons for this including lack of support, course difficulty, inappropriate learner expectations, lack of time, and poor technology skills (Onah ref).

Nonetheless, MOOCs have been celebrated for their inclusivity, allowing learners flexibility to choose when to take part in learning, providing easy incorporation of accessibility tools such as image captioning, and allowing learners across the globe to access materials (Lambert, 2020).

## 3 VARIANT INTERPRETATION MOOC DESIGN AND DELIVERY

Two MOOCs were designed through the CanGene‐CanVar research program (CanGen‐CanVar, 2022) which address both core principles in rare disease genomics and the intricacies of cancer susceptibility gene variant interpretation:

- Interpreting Genomic Variation: Fundamental Principles (FP)
- Interpreting Genomic Variation: Inherited Cancer Susceptibility (ICS)

The target audience for these MOOCs are HCPs involved in genomic testing, including clinical scientists, genetic counselor and doctors across specialties.

A working group of genomics specialists including cancer and clinical genetics consultants, a genetic counselor, genetics specialist registrars (SpRs), clinical scientists and a chartered health psychologist led the MOOC design. Intended Learning Outcomes were drafted to guide curriculum design for each course (See Box 1).

BOX 1 **Intended learning outcomes**

**Interpreting genomic variation: Fundamental principles**

After completing this course, the learner should be able to:

- Describe the different types of genomic variants and interpret the impact of different variant types in the context of normal background genomic variation;
- Apply the tools used in variant classification (including population databases, inheritance data, predictive data, functional data and phenotype) to interpret genomic variation;
- Critically appraise the strengths and weaknesses of each of the tools available for variant interpretation;
- Apply the American College of Medical Genetics and Genomics (ACMG) variant interpretation framework, including updates from the Association for Clinical Genomic Science (ACGS), to classify variants in rare disease genes;
- Appreciate the value of the multidisciplinary team (MDT) approach in ensuring high‐quality variant interpretation and patient care, and learn how to communicate efficiently in an MDT setting.

**Interpreting genomic variation: Inherited cancer susceptibility**

After completing this course, the learner should be able to:

- Critically appraise the strengths and weaknesses of the American College of Medical Genetics (ACMG) guidelines for variant interpretation in complex disease, such as cancer, compared to rare pediatric disease
- Describe the importance of large‐scale collaborative infrastructure to standardize evidence based variant interpretation in cancer susceptibility genes
- Apply the CanVIG‐UK guidelines for variant interpretation to classify variants in cancer susceptibility genes involved in hereditary breast and ovarian cancer and Lynch Syndrome
- Critically appraise the complexities of applying cancer susceptibility gene variant interpretation in clinical practice including the implications of reduced penetrance variants and potential changes in classifications over time
- Consider the different ways in which cancer susceptibility gene variant interpretation can impact on patient care

To structure learning, the FP course considers the foundational knowledge required for robust variant interpretation, including the ACMG and ACGS guidance (Ellard et al., 2020; Richards et al., 2015). The ICS course builds on this, utilizing the CanVIG‐UK guidance for cancer susceptibility gene variant interpretation (Garrett et al., 2021).

A continuous feedback process was used to create a broad curriculum mapped to a series of activities over several weeks (See Table 1). Each week expected to take learners 3–4 h to complete, however, all content is released to learners upon registration allowing flexibility to set their own pace.

Care was taken to provide both procedural and conceptual scaffolding for learners (Jumaat & Tasir, 2014) A core focus across both courses were the fundamental concepts of variant interpretation, thus providing a foundational knowledge for learners to build upon, given the ever‐changing nature of our understanding of genomic variant interpretation and the guidelines utilized for this. To then support a procedural understanding multiple quizzes and exercises were used to allow learners to practice using various variant interpretation tools.

**FIGURE 1:**
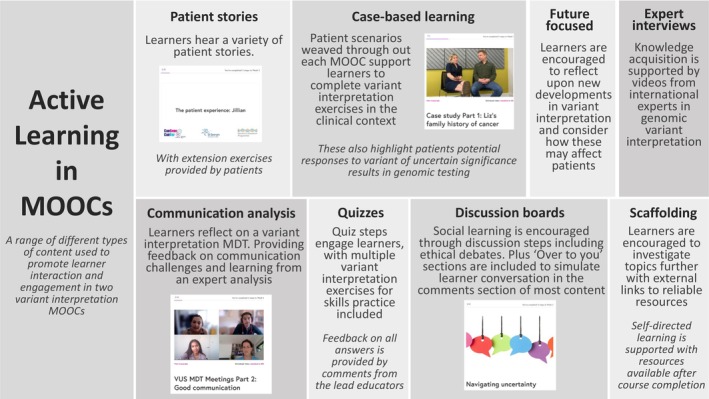
Examples of active learning approaches used in the content of both MOOCs.

Content design was guided by active learning pedagogies in online education, including Mayer's Multimedia Principles (Mayer & Moreno, 2005). This content was created by members of the working group, contributing mixed media steps for each course including text‐based articles, videos, recorded presentations, quizzes and discussion boards. Examples of the active learning approaches used can be seen in Figure 1. A central aspect of both courses was the case‐based approach, which has been used with success in other healthcare MOOCs (Schettino & Capone, 2022). A fabricated patient case was weaved throughout the narrative of both the FP and ICS course to highlight clinical relevance, and to create opportunities for learner interaction and enhance learner engagement.

Content was rigorously reviewed both for accuracy by relevant scientific experts, and by education specialists to ensure Mayer's principles were applied throughout. This included a focus on relevant images, graphics and spoken word to support text‐based content, the use of a conversational voice throughout and a focus on coherence and minimal redundancy of information (Mayer & Moreno, 2005). Beyond this several considerations were made for the online learning accessibility with all content including features such as video transcripts, alternate text to describe images and resources formatted for use with screen readers (Burgstahler, 2021).

The FutureLearn MOOC platform (www.futurelearn.com) was used to host the courses. Our institution has an existing contract with FutureLearn to develop MOOCs on this platform, and the course creators had prior positive experiences with this platform. FutureLearn has also been used successfully for other genomics MOOCs in the literature (Bishop et al., 2019).

The iterative process of curriculum design, content creation and expert review took approximately 12 months, with both courses launching on 10th January 2021. Initially an evaluation cohort was recruited to provide detailed feedback. This cohort included 94 genomics specialist HCPs (30 Genetic Counselors, 44 Clinical genetics trainees or consultants and 20 Clinical Scientists) and 39 clinicians working outside of clinical genetics who order genomic testing (10 Oncologists, 8 other cancer clinicians, 4 hematologists, 7 Pediatricians, 1 Neurologist and 9 cardiologists). Participants were asked to complete a series of questionnaires about the courses at various timepoints, with their performance on the course also analyzed.

While the full evaluation will be published elsewhere (Coad et al., 2023), below we present a selection of findings to consider the utility of this education intervention in the genetic counseling workforce.

## 4 GENETIC COUNSELOR EVALUATION OF MOOCs

Genetic counseling participants were recruited through professional body and training program email groups, with 30 genetic counselors at different stages of their career agreeing to take part in the evaluation (Trainee/Student = 11/30, Preregistration = 7/30, Registered = 12/30) (Middleton et al., 2022). To complete the full evaluation participants were required to work through ≥90% of the course materials, and return both pre‐ and postcourse questionnaires within a 3‐month timeframe. A 57% (17/30) completion rate was seen amongst genetic counselors (See Table 2).

**TABLE 2:** Number of genetic counselors at different levels of experience completing the full evaluation.

Course (s) completed	Experience level			Totals
	Registered	Preregistration	Trainee/ student	
FP only	1	2	1	4
ICS only	1	0	0	1
Both	5	2	5	12
Totals	8	4	6	17

*Note*: Some participants may have chosen to only complete the FP or ICS course due to their prior learning or relevance to their clinical role.

**TABLE 1:** Curriculum map showing the activity sections for both the FP and ICS MOOC.

Interpreting genomic variation: FP		
Week one	Week two	Week three
Introduction	Computational and predictive data	ACMG & ACGS guidelines
Normal genetic variation	Functional data	VUS multidisciplinary meetings
Changing technologies	Reputable databases	
Variant classification	Phenotyping and literature searches	Genomic variant interpretation in practice
Population databases	Ethics of genomic variant interpretation	

Interpreting genomic variation: ICS	
Week one	Week two
Introduction	Functional data
Differences in cancer susceptibility genes	Allelic data
Adapting the ACMG framework	Reputable sources
Population data	Other data
	Other consideration
Computational and predictive data	In practice
	The evolving landscape

Abbreviations: ACMG, American College of Medical Genetics and Genomics; ACGS, Association for Clinical Genomic Science; FP, Fundamental Principles; ICS, Inherited Cancer Susceptibility; MOOC, Massive Open Online Course.

Completion rates were similar between Trainees/MSc Students (55% *n* = 6/11) and Preregistration Genetic Counselors (57% *n* = 4/7), with a slightly higher completion rate seen for Registered Genetic counselors (66% *n* = 8/12). To consider the reason for attrition, those participants who did not finish the full evaluation were asked to complete a short questionnaire to highlight reasons for this. Of the 13 genetic counselors who did not complete the evaluation, four responded (Trainee/Student = 2, Preregistration = 2) clarifying that they did not have enough time to complete the course due to work commitments.

### 4.1 Pre‐ and postcourse confidence

Genetic counselors were asked to rate their confidence across different aspects of variant interpretation on a Likert scale (see Data S1 and S2 for questionnaires). Across all levels of experience average confidence scores improved, with greater improvements seen in the ICS course (FP Trainee/Student = +1.13, Preregistration = +0.82, Registered = +0.67) (ICS Trainee/Student = +1.47, Preregistration = +1.79, Registered = +1.10).

### 4.2 Postcourse feedback

**FIGURE 2:**
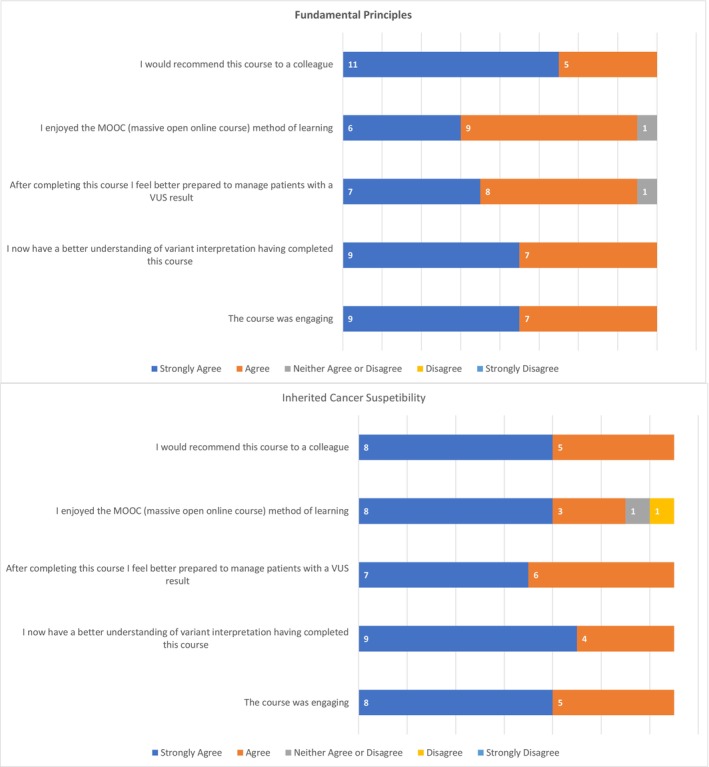
Summary of genetic counselor responses to a selection of five statements about the MOOCs.

In addition to the confidence scores, the postcourse questionnaire included a series of 20 questions, 10 of which asked if participants agreed with statements about learner satisfaction, perceived knowledge gained and utility in clinical practice, utilizing a Likert scale for responses (see Data S1 and S2 for questionnaires). All genetic counselors who completed the evaluation gave responses about the relevant MOOC (FP *n* = 16, ICS *n* = 13). See Figure 2 for a summary of responses to a subset of five of these questions.

Despite the courses design for a broad range of workforces, most genetic counselors (FP 94% *n* = 15/16, ICS 100% *n* = 13/13) agreed that learning from this course would impact their clinical practice. The MOOC learning style was also well received by genetic counselors who mostly enjoyed this method of learning (FP 94% *n* = 15/16, ICS 85% *n* = 11/13).

As well as the 10 Likert scale questions, participants were asked four further multiple‐choice questions and six free text questions to gather more detailed feedback specific to their role (see Data S1 and S2 for questionnaires). All genetic counselors who completed the evaluation gave responses to these additional questions (FP *n* = 16, ICS *n* = 13). A summary of responses is outlined below.

The step‐by‐step quizzes going through variant classification were extremely useful.–Student Genetic Counsellor

Genetic counselors frequently highlighted the utility of working through case examples in both the FP (*n* = 9) and ICS (*n* = 10) courses. Overall comments about these quiz steps where very positive, however, some highlighted difficulties when using external databases (FP *n* = 3 ICS *n* = 6). While this highlights an additional training need, a likely explanation for this was that many genetic counselors did not have access to preferred variant interpretation software. This could be due to limited license availability in departments, or database holders lack of understanding for genetic counselors' central role as part of the genomic variant interpretation MDT.

I felt it was very helpful for me personally as a Genetic Counsellor to recap and improve on variant interpretation.–Pre‐Registered Genetic Counsellor

The genetic counselors had different levels of experience in variant interpretation, this was highlighted in the free text sections with some describing themselves as ‘novices’ or new to certain areas (*n* = 3), while others highlighted sections of the course where particularly easy and a review of existing knowledge (*n* = 3). No participants stated they knew all information in either course, however, fewer genetic counselors stated they knew most of the information in the ICS course (FP 50% *n* = 8/16, ICS 23% *n* = 3/13). Despite varying levels of experience in variant interpretation, the majority of genetic counselors felt they would use learning from the course on at least a weekly basis (FP 69% *n* = 11/16, ICS 58% *n* = 7/12).

More interaction is always a bonus–Registered Genetic Counsellor

Some participants did highlight a desire for more interaction or further feedback from the course (FP = 3, ICS = 4). Some learners (FP = 3, ICS = 3) also indicated that working through the materials alone was difficult, suggesting a potential benefit for some synchronous content delivery.

## 5 DISCUSSION

These first of their kind FP and ICS MOOCs received positive feedback from genetic counselors, suggesting these MOOCs could be a useful tool in educating this specialists workforce in genomic variant interpretation. Interestingly, participants felt they had more prior knowledge in the FP topics than ICS, which may reflect the fast‐moving nature of guidance updates in cancer susceptibility gene variant interpretation (Garrett et al., 2021).

### 5.1 Limitations

While some learners did report a lack of interaction during these MOOCs, this may have been due to the limited interaction possible between the few participants in the early run of these courses before they had been more widely advertised to a larger audience of learners. This is consistent with the broader literature, where a lack of interaction and needs for greater support were noted as potential reasons for high attrition rates across MOOCs (Onah et al.). This MOOC also saw significant attrition as is many healthcare MOOCs (Liyanagunawardena & Williams, 2014; Schettino & Capone, 2022), however our study found the core reason for noncompletion being lack of learner time, as also highlighted by Onah et al. (2014).

While an increase in learner confidence was seen in these MOOCs, as with other healthcare MOOCs, the impact of this on clinical practice was not within the scope of this study (Bishop et al., 2019; Cao et al., 2021; Hoedebecke et al., 2018; Hossain et al., 2015; Magaña‐Valladares et al., 2018).

These finding only represent a small self‐selecting sample of UK based genetic counselors. This small sample size means that the data did not have adequate power for robust statistical analysis, and that these results may not be representative of the workforce as a whole. In addition, due to the limitations of the MOOC platform, wider data around knowledge gained and skills acquired by participants is not available.

### 5.2 Future work

The feedback from learners in this evaluation will be used to improve the MOOCs content. The courses will then be advertised more widely through professional social media channels and international conferences to improve access, and provide an opportunity to gain feedback from a greater breadth of the workforce. Combining the MOOCs with social media could also serve to improve course completion rates as illustrated by Hoedebecke et al. (2018).

To address the limited interaction highlighted by learners a blended approach could be applied, including workshops where learners can tackle challenging areas together as highlighted by Jia et al. (2019). Alternatively, an approach similar to Bishop et al. (2019) could be used, in which MOOC runs are supported by specialist tutors.

Beyond this, future studies would be required to measure the impact of this training on genomic counselors' clinical practice, considering how they may apply the knowledge learned over an extended time period.

## 6 CONCLUSIONS

The MOOC education style does hold huge potential for the future of genomic variant interpretation training. Providing flexible learning opportunities with a wide reach which could meet the challenges of keeping the genetic counseling workforce up to date with the ever‐evolving guidance in this field, supporting safe management of patients with genomic test results.

## AUTHOR CONTRIBUTIONS

The manuscript was drafted by BC with detailed review by KS. AF, BC, KJ, KS, KT‐B and AF contributed to MOOC and evaluation design, AR supported data analysis. All authors read and approved the final manuscript. BC and KS confirm that they had full access to all the data in the study and take responsibility for the integrity of the data and the accuracy of the data analysis. All of the authors gave final approval of this version to be published and agree to be accountable for all aspects of the work in ensuring that questions related to the accuracy or integrity of any part of the work are appropriately investigated and resolved.

## CONFLICT OF INTEREST STATEMENT

All authors declare that they have no conflict of interest.

## ETHICS STATEMENT

Human studies and informed consent: This study was conducted in accordance with all guidelines set forth by St George's University of London. All participants provided informed consent to participate in this evaluation, and St George's University Of London waived the need for formal ethics approval for use of the anonymized data in this evaluation.

Animal studies: No nonhuman animal studies were carried out by the authors for this article.

## Supporting information


Data S1.

Data S2.

## Data Availability

The datasets used and/or analyzed during the current study are available from the corresponding author on reasonable request.
