# Supplementary material for: Massive open online courses (MOOCs) in genomic variant interpretation: An innovative education strategy for the growing genetic counselor workforce
Source: J Genet Couns. 2023 Nov 27;33(1):142–50. doi: 10.1002/jgc4.1837 (PMC11170928; doi:10.1002/jgc4.1837)
Supplement: Supplementary file 1 — Data S1. [file JGC4-33-142-s002.pdf]

1b) Interpreting Genomic Variation: Fundamental Principles- Post course

This short quiz is to be taken by all evaluators once they have completed the Interpreting Genomic Variation: Fundamental Principles

* Required

1. Please enter the email address you used to sign up to the course: *

2. Having completed the 'Interpreting Genomic Variation: Fundamental Principles' course, on a scale of 1-5 how confident would you feel to perform the following: *

	Not confident at all	Slightly confident	Somewhat Confident	Quite confident	Extremely confident
Explain what a variant of uncertain significance (VUS) is to a patient/relative/colleague	○	○	○	○	○
Discuss and debate the appropriate use of	Not confident at all	Slightly confident	Somewhat Confident	Quite confident	Extremely confident

evidence from population databases in genomic variant interpretation	○	○	○	○	○
Discuss and debate the appropriate use of evidence from computational and predictive data in genomic variant interpretation	○	○	○	○	○
Discuss and debate the likely pattern of inheritance in a family and how this can be used in genomic variant interpretation	○	○	○	○	○
Discuss and debate the appropriate use of evidence from functional data and how this can be used in genomic variant interpretation	○	○	○	○	○
Discuss and debate the appropriate use of phenotype information	Not confident at all	Slightly confident	Somewhat Confident	Quite confident	Extremely confident

and how this can be used in genomic vari ant interpretation	◯	◯	◯	◯	◯
Appraise the strengths and weakness of different lines of evidence available for genomic variant interpretation	◯	◯	◯	◯	◯
Participate in a discussion of genomic variant interpretation at an MDT meeting	◯	◯	◯	◯	◯
Apply the ACMG guidelines to the classification of genomic variants	◯	◯	◯	◯	◯
Apply the ACGS guidelines to the classification of genomic variants	◯	◯	◯	◯	◯
Communicate a VUS result to a patient/relati ve/colleague	◯	◯	◯	◯	◯
Explain to a patient/relati ve/colleague what additional information	Not confident at all ◯	Slightly confident ◯	Somewhat Confident ◯	Quite confident ◯	Extremely confident ◯

[illegible] or testing may be needed to classify a variant	◯	◯	◯	◯	◯
Explain why variant interpretation in complex disease, like cancer, has different considerations than variant interpretation in rare disease	◯	◯	◯	◯	◯
Explain to a patient/relative/colleague how the classification of a genomic variant may change over time	◯	◯	◯	◯	◯

3. To what extent do you agree or disagree with the following statements about the 'Interpreting Genomic Variation: Fundamental Principles' course: *

	Strongly disagree	Disagree	Neither agree nor disagree	Agree	Strongly Agree
The course was engaging	◯	◯	◯	◯	◯
I had opportunities to interact with other learners	◯	◯	◯	◯	◯
This course has improved my confidence [illegible]	◯	◯	◯	◯	◯

Strongly disagree Disagree Neither agree nor disagree Agree Strongly Agree

with genomic variant interpretation

	Strongly disagree	Disagree	Neither agree nor disagree	Agree	Strongly Agree
I now have a better understanding of variant interpretation having completed this course	○	○	○	○	○
Learning from this course will impact on my clinical practice	○	○	○	○	○
The course was relevant for my professional development	○	○	○	○	○
After completing this course I feel better prepared to engage in MDT meetings where VUS are discussed	○	○	○	○	○
After completing this course I feel better prepared to manage patients with a VUS result	○	○	○	○	○
I enjoyed the MOOC (massive open online course) method of learning	○	○	○	○	○

I would

I would recommend this course to a colleague ◯ ◯ ◯ ◯ ◯

4. Approximately what proportion of the content in the 'Interpreting Genomic Variation: Fundamental Principles' course was new to you? *

◯ 0%- I knew everything that was provided on the module

◯ 25%- I knew most of the information

◯ 50%- I knew some information, but a lot was new to me

◯ 75% - I knew the basic information, and most of the information was new to me

◯ 100%- I did not know any or only minimal information and the majority of the information was new to me

5. How often will you use the content you have learnt in 'Interpreting Genomic Variation: Fundamental Principles' in a clinical context? For example, in patient contact in clinic, in multi-disciplinary meetings, or in the interpretation of genomic test results. *

◯ Every day

◯ Every week

◯ Every month

◯ At least once a year

◯ Rarely (less than once a year) or never

6. From your perspective, was the content in this course *

◯ Too complex

◯ About the right level

◯ Too simple

7. Considering just the time you spent learning on the course, how long on average did it take you to complete all the steps in each week of the course? *

- ◯ Less than 3 hours
- ◯ 3-5 hours
- ◯ 5-10 hours
- ◯ Over 10 hours

8. What did you enjoy most about the course? *

9. What did you enjoy least about the course? *

10. Was there any information you felt was missing from the course? *

11. Was there any part of the course you found particularly easy? *

12. Was there any part of the course you found particularly difficult? *

13. How could we improve the course? *

This content is neither created nor endorsed by Microsoft. The data you submit will be sent to the form owner.

Microsoft Forms
