# Supplementary material for: Massive open online courses (MOOCs) in genomic variant interpretation: An innovative education strategy for the growing genetic counselor workforce
Source: J Genet Couns. 2023 Nov 27;33(1):142–50. doi: 10.1002/jgc4.1837 (PMC11170928; doi:10.1002/jgc4.1837)
Supplement: Supplementary file 2 — Data S2. [file JGC4-33-142-s001.pdf]

2b) Interpreting Genomic Variation: Inherited Cancer Susceptibility- Post course

This short quiz is to be taken by all evaluators once they have completed the Interpreting Genomic Variation: Inherited Cancer Susceptibility

* Required

1. Please enter the email address you used to sign up to the course: *

2. Having completed the 'Interpreting Genomic Variation: Inherited Cancer Susceptibility' course, on a scale of 1-5 how confident would you feel to perform the following: (CSG = Cancer Susceptibility Gene) *

	Not confident at all	Slightly confident	Somewhat Confident	Quite confident	Extremely confident
Explain what a variant of uncertain significance (VUS) is to a patient/relative/colleague	○	○	○	○	○
Discuss and debate the appropriate	Not confident at all	Slightly confident	Somewhat Confident	Quite confident	Extremely confident

	Not confident at all	Slightly confident	Somewhat Confident	Quite confident	Extremely confident
[illegible] use of evidence from population databases in variant interpretation in CSGs	○	○	○	○	○
Discuss and debate the appropriate use of evidence from computational and predictive data in variant interpretation in CSGs	○	○	○	○	○
Discuss and debate the likely pattern of inheritance in a family and how this can be used in CSG variant interpretation	○	○	○	○	○
Discuss and debate the appropriate use of evidence from functional data and how this can be used in CSG variant interpretation	○	○	○	○	○
Discuss and debate the appropriate use of phenotype information and how this can be used in	○	○	○	○	○

	Not confident at all	Slightly confident	Somewhat Confident	Quite confident	Extremely confident
[illegible] CSG variant interpretation					
Appraise the strengths and weakness of different lines of evidence available for genomic variant interpretation in CSGs	○	○	○	○	○
Participate in a discussion of CSG variant interpretation at an MDT meeting	○	○	○	○	○
Explain why variant interpretation in complex disease, like cancer, has different considerations than variant interpretation in rare disease	○	○	○	○	○
Apply the CanVIG-UK guidance to the classification of genomic variants in cancer susceptibility genes	○	○	○	○	○
Communicate a CSG VUS result to a patient/relative/colleague	○	○	○	○	○
Explain to a patient/relati[illegible]					

patient/relative/colleague what additional information or testing may be needed to classify a variant	○	○	○	○	○
Use the evidence points system to refine more clearly the probability that a variant is pathogenic	○	○	○	○	○
Explain to a patient/relative/colleague how the classification of a genomic variant may change over time	○	○	○	○	○

3. To what extent do you agree or disagree with the following statements about the 'Interpreting Genomic Variation: Inherited Cancer Susceptibility' course: *

	Strongly disagree	Disagree	Neither agree nor disagree	Agree	Strongly Agree
The course was engaging	○	○	○	○	○
I had opportunities to interact with other learners	○	○	○	○	○
This course has improved my	Strongly disagree	Disagree	Neither agree nor disagree	Agree	Strongly Agree

	Strongly disagree	Disagree	Neither agree nor disagree	Agree	Strongly Agree
confidence with genomic variant interpretation	◯	◯	◯	◯	◯
I now have a better understanding of variant interpretation having completed this course	◯	◯	◯	◯	◯
Learning from this course will impact on my clinical practice	◯	◯	◯	◯	◯
The course was relevant for my professional development	◯	◯	◯	◯	◯
After completing this course I feel better prepared to engage in MDT meetings where VUS are discussed	◯	◯	◯	◯	◯
After completing this course I feel better prepared to manage patients with a VUS result	◯	◯	◯	◯	◯
I enjoyed the MOOC (massive open online course) method of learning	◯	◯	◯	◯	◯

I would recommend this course to a colleague	◯	◯	◯	◯	◯
I would not have been able to complete the 'Inherited cancer predisposition course' without first completing the 'fundamental principles' course	◯	◯	◯	◯	◯

4. Approximately what proportion of the content in the 'Interpreting Genomic Variation: Inherited Cancer Susceptibility' course was new to you? *

◯ 0%- I knew everything that was provided on the module

◯ 25%- I knew most of the information

◯ 50%- I knew some information, but a lot was new to me

◯ 75% - I knew the basic information, and most of the information was new to me

◯ 100%- I did not know any or only minimal information and the majority of the information was new to me

5. How often will you use the content you have learnt in 'Interpreting Genomic Variation: Inherited Cancer Susceptibility' in a clinical context? For example, in patient contact in clinic, in multi-disciplinary meetings or genomic tumour advisory boards or in the interpretation of genomic test results. *

- ◯ Every day
- ◯ Every week
- ◯ Every month
- ◯ At least once a year
- ◯ Rarely (less than once a year) or never

6. From your perspective, was the content in this course *

- ◯ Too complex
- ◯ About the right level
- ◯ Too simple

7. Considering just the time you spent learning on the course, how long on average did it take you to complete all the steps in each week of the course? *

- ◯ Less than 3 hours
- ◯ 3-5 hours
- ◯ 5-10 hours
- ◯ Over 10 hours

8. What did you enjoy most about the course? *

9. What did you enjoy least about the course? *

10. Was there any information you felt was missing from the course? *

11. Was there any part of the course you found particularly easy? *

12. Was there any part of the course you found particularly difficult? *

13. How could we improve the course? *

This content is neither created nor endorsed by Microsoft. The data you submit will be sent to the form owner.

Microsoft Forms
